# Visual Sensorial Impairments in Neurodevelopmental Disorders: Evidence for a Retinal Phenotype in Fragile X Syndrome

**DOI:** 10.1371/journal.pone.0105996

**Published:** 2014-08-25

**Authors:** Rafaëlle Rossignol, Isabelle Ranchon-Cole, Arnaud Pâris, Ameziane Herzine, Astrid Perche, David Laurenceau, Pauline Bertrand, Christine Cercy, Jacques Pichon, Stéphane Mortaud, Sylvain Briault, Arnaud Menuet, Olivier Perche

**Affiliations:** 1 UMR7355, CNRS, Orléans, France; 2 Experimental and Molecular Immunology and Neurogenetics, University of Orléans, Orléans, France; 3 Genetic Department, Regional Hospital, Orléans, France; 4 Laboratory of Sensorial Biophysical, University of Clermont 1, Clermont-Ferrand, France; CNRS UMR7275, France

## Abstract

Visual sensory impairments are common in Mental Deficiency (MD) and Autism Spectrum Disorder (ASD). These defects are linked to cerebral dysfunction in the visual cortical area characterized by the deregulation of axon growth/guidance and dendrite spine immaturity of neurons. However, visual perception had not been addressed, although the retina is part of the central nervous system with a common embryonic origin. Therefore, we investigated retinal perception, the first event of vision, in a murine model of MD with autistic features. We document that retinal function is altered in *Fmr1* KO mice, a model of human Fragile X Syndrome. Indeed, In *Fmr1* KO mice had a lower retinal function characterized by a decreased photoreceptors neuron response, due to a 40% decrease in Rhodopsin content and to Rod Outer Segment destabilization. In addition, we observed an alteration of the visual signal transmission between photoreceptors and the inner retina which could be attributed to deregulations of pre- and post- synaptic proteins resulting in retinal neurons synaptic destabilization and to retinal neurons immaturity. Thus, for the first time, we demonstrated that retinal perception is altered in a murine model of MD with autistic features and that there are strong similarities between cerebral and retinal cellular and molecular defects. Our results suggest that both visual perception and integration must be taken into account in assessing visual sensory impairments in MD and ASD.

## Introduction

Mental Deficiency (MD) and Autism Spectrum Disorders (ASD) are frequent pathologies (more than 2% of worldwide population [1], [2]) appearing during childhood. They are both characterized by impairments in cognitive functions, social integration, and/or communication. Apart from this “core” deficit, hallmark for diagnosis of MD and ASD, patients frequently demonstrated a range of sensory abnormalities [1], [3]–[5]. Indeed, atypical responses to sensory stimulation have been reported in approximately 70–90% of individuals with MD or ASD [1], [6]–[9]. Sensory impairment is defined as the inability to interpret outside stimuli such as visual, auditory, verbal, sense of touch, taste, smell or feeling pain, and may manifest as both hyper and hyposensitivity to stimulation. Among hypothesis underlying the neurophysiological basis of such impairments, the mis-wiring of neuronal connections in the developing brain and synaptic destabilization had been reported. Indeed, MD or ASD have been linked to cerebral dysregulation of axon growth/guidance and dendrite spine maturation [10]–[12] leading to synaptic defects [13]–[15]. Currently, sensory impairments are attributed to a cerebral phenotype.

Fragile X Syndrome (FXS) is the most common form of MD with ASD features [16], [17]. This X-linked disorder is caused by the absence of FMRP (Fragile X Mental Retardation Protein) due to the transcriptional silencing of *FMR1* gene (*Fragile X Mental Retardation gene 1*). This FMRP defect leads to a large number of synaptic proteins alterations [18],[19] and consequently to neuronal dendrite spine immaturity and synaptic impairment at brain level [19]–[21]. It has been shown that vision integration is particularly affected in FXS patients, with alteration of spatiotemporal visual processing, reduction of contrast sensitivity for visual stimuli presented at high temporal frequencies, and visual sensitivity for both static (texture difference) and moving images [22], [23]. These defects in visual sensory were associated with cerebral neuron immaturity [12], [15] especially in primary visual cortex [11].

However, before being integrated at the cerebral level, the visual signal has to be detected by- and transmitted through- the retina. At this level, the physical signal, light, is transformed into an electrophysiological signal by highly specialized cells, the photoreceptors. The electrophysiological signal is then transmitted and modulated through the retina before reaching the optic nerve to the brain. So far, no data have been collected on light perception by the retina in MD, ASD or FXS. Despites its peripheral location, the retina is part of the central nervous system and shares a common neurodevelopmental origin with the diencephalon [24], [25]. Therefore, the retina presents a strong similarity with brain neurons in terms of neurotransmitters, composition in highly differentiated neurons and functional processes [26]. Based on these similarities, we hypothesized that the retinal perception itself might also be altered.

In order to address this question, we investigated retinal features in a murine model of MD with autistic features, the *Fmr1* KO mice, model of Fragile X Syndrome. This mouse is considered as a relevant model of FXS since it presents behavioral, cellular and molecular phenotypes similar to human FXS [12], [27]–[29]. Therefore, in the *Fmr1* KO mice, we studied whether the absence of Fmrp expression could affect retinal structure, function and molecular markers of neurotransmission.

## Methods

### Animals

Male *Fmr1* Knock-Out (KO) and their wild-type (WT) littermates (adult: 6-months-old) were generated by breeding heterozygous *Fmr1* ^+/−^ females with C57BL/6J background WT males [28]. Mice were weaned at 21 days of age and group-housed with their same-sex littermates. On the same day, tail samples were collected for DNA extraction and for subsequent PCR assessment of genotypes as previously described [28], [30]. Food and water were provided *ad libitum*. Animals were maintained under temperature (22°C) and humidity (55%) controlled conditions with a 12∶12 hr dim light–dark cycle (25 lux, lights on at 7 a.m.). For tissue collection, mice were euthanized by CO_2_ gas inhalation or anesthetized by a single intraperitoneal injection of 44 mg/kg ketamine and 8 mg/kg xylazine in saline followed by cervical dislocation. The present experimental protocol received full review and approval by the regional animal care and use committee (Comité Régional d’Ethique à l’Expérimentation Animale - CREEA) prior to conducting the experiments.

### Electroretinography

After overnight dark adaptation, mice were anesthetized with ketamine (50 mg/kg) and xylazine (2 mg/kg). Eye drops were used to dilate the pupil (Atropine sulfate 1%, ALCON). Mice were placed on a temperature-regulated heating pad throughout the recording session. Strobe flash ElectroRetinoGrams (ERGs) (10 µs) were recorded using an Ag/AgCl electrode in contact with the corneal surface. An Ag/AgCl electrode was placed on the tong and a copper reference screen under the animal. Dark-adapted responses were presented within an integrating sphere (Labsphere, France) that mimics a ganzfeld and allows to illuminate uniformly the all retina. ERGs are recorded using flash intensities ranging from −3.47 to +0.46 log cd s/m^2^. Stimuli were presented in order of increasing intensity. Conversely, the duration of the interstimulus interval is 30 s since this interval has been shown to be sufficient for a flash not to alter the next flash response. Responses were differentially amplified (0.3–10,000 Hz), averaged, and stored. Intensity–response functions were obtained in a single session.

### ERG analysis

The leading edge of the a-waves obtained in response to the highest intensity stimuli was analyzed with a modified form of the Lamb–Pugh model of rod phototransduction [31]–[33] equation: P3 = {1−exp[−*i*S*_A_*(*t*−*t*_d_)^2^]}A_max_ (1) where P3 represents the massed response of the rod photoreceptors and is analogous to the PIII component of Granit [31]. The amplitude of P3 is expressed as a function of flash energy (*i*) and time (*t*) after flash onset. S*_A_* is the gain of phototransduction, *A*_max_ is the maximum response, and *t*_d_ is a brief delay. The amplitude of the b-wave is calculated from the minimum of the a-wave to the maximum of the b-wave. Intensity–response function of the b-wave amplitude was fitted with the Naka–Rushton equation: *B*/*B*_max_ = I*^n^*/(I*^n^*+*K^n^*) (2) where *I* is the stimulus luminance of the flash (2,88 cd.s.m^−2^), *B* is the b-wave amplitude of ERG at *I* luminance, *B*_max_ is the asymptotic b-wave amplitude, *K* is the half-saturation constant corresponding to retinal sensitivity and *n* is a dimensionless constant controlling the slope of the function. The latency is the time interval between the stimulation and the peak of the b-wave or the a-wave. Oscillatory Potentials (OPs) were recorded by using a band-pass between 30 and 300 Hz. For each OPs, the amplitude from the baseline to the peak and the latency were calculated.

### Rod response recovery

To evaluate rod response recovery after bleaching, a single test flash of 2.88 (cd.s.m^−2^) was presented on dark-adapted retina, then mice were exposed to a steady light for 2 min to bleach the retina. Immediately after bleaching and then every 10 min for 90 min, a single test flash of 2.88 (cd.s.m^−2^) was presented. The a-wave response at the indicated time after bleaching was normalized to the initial dark-adapted response for each mouse.

### Quantitative RT-PCR and Western Blotting

Quantitative RT-PCR, performed using Taqman technologies (Applied technologies), and Western blotting were realized as described previously [34], [35]. Further experimental details were described in Supporting Information S1.

### Retinal Histology and Golgi staining

Retinal histology was done as described previously [36]. Retinal layers thicknesses were measured every 0.78 mm from the optic nerve to the inferior and to the superior ora serrata.

A modified Golgi staining method based on FD Rapid GolgiStain Kit (FDNeuroTechnologies, Ellicott, USA) was used. WT and *Fmr1* KO eyes cup were dissected in 4% PFA pH 7.4, and cornea and lens were removed. Eye cup was incubated in the dark for 2 days (room temperature) in impregnation solution of the kit, and then placed 1 day in post-fixative solution (in the dark, room temperature). Free-floating 150 µm vibratome retinal sections obtained from eye cup were cut along the meridian through the optic nerve. Sections were incubated 30 minutes at room temperature in revelation solution of the kit. Retinal sections were dehydrated in graded ethanol, cleared in xylene, and mounted in paramount (Fisher Scientific). Every 100 µm of retina from the optic nerve to the inferior and to the superior ora serrata, number of neurons with or without exuberant and disorganised dendrites was counted under microscope (x400, Leica, Paris, France). Mean values in each group were presented as percentage of WT.

### Rhodopsin Quantification

Rhodopsin quantification was realized as described before [37]. We measured total Rhodopsin levels by spectral analysis (300–700 nm) using the differential absorbance of Rhodopsin at 498 nm before and after bleaching (10 min). Whole eyes were homogenized in 240 µL ROS buffer (1 mM HEPES, pH 7.4; 3 mM NaCl; 6 mM KCl; 0.2 mM MgCl2; 0.1 mM DTT) supplemented with 50 mM hydroxylamine, 1.5% maltoside and proteinase inhibitor cocktail (Pierce, Paris, France). The samples were spun down at 3000 g for 5 min at 4°C and the supernatant was assayed. Rhodopsin concentration was calculated by difference absorbance at 498 nm using the molar extinction coefficient of 42,700 M^−1^.cm^−1^.

### PSD95, Syt1a, mGluR5 and Rhodopsin immunohistochemistry

PSD95, Syt1a and mGluR5 immunochemistry were done on eye cryosections (14 µm), whereas Rhodopsin was tested on paraffin sections (4 µm). Briefly, sections were heated 30 minutes in sodium citrate buffer pH 6.0 and then blocked 2 hours in blocking buffer (10% FBS, 2% BSA, 0.3% Triton X-100 and 0.1% NaN_3_) at room temperature. Sections were incubated with a 1∶500 dilution of PSD95, Syt1a, mGluR5 or Rhodopsin antibodies (Abcam, Paris, France) overnight at 4°C in blocking buffer. After washing them three times in TBS pH 7.6, sections were stained with 1∶100 dilution goat anti-mouse secondary antibody (Life Technologies, Carlsbad, USA) (FITC for PSD95, Syt1a and mGluR5 and TRITC for Rhodopsin). DAPI at 10 µg/mL (Roche Applied Science, Indianapolis, USA) was then applied on sections for 10 minutes at room temperature. Sections were washed three times in TBS pH 7.6, mounted with Fluoromount (Vector Laboratories, Burlingame, USA) and stored at 4°C. Imaging was realised on a Carl Zeiss apotome (40X, 40/1.3) and analysed on AxioVision software thank to the Cytometry and Imagery platform of the CBM (Orléans, France).

### Electron microscopy

Electron microscopy was realized in Centre d’Imagerie Cellulaire Santé (Clermont-Ferrand, France). Experimental procedure is described in Supporting Information S1.

### Statistical analysis

All results are expressed as mean ± SEM. Data analysis was performed using GraphPad Prism 6.00. Statistical comparisons among groups were conducted using Student’s unpaired *t* test. Statistical significance was defined as *p*<0.05. Significant differences between groups are noted by *. One symbol for p<0.05; two symbols for p<0.01; three symbols for p<0.001; four symbols for p<0.0001.

## Results

### Fmrp is expressed in WT mice retinas

**Figure 1:**
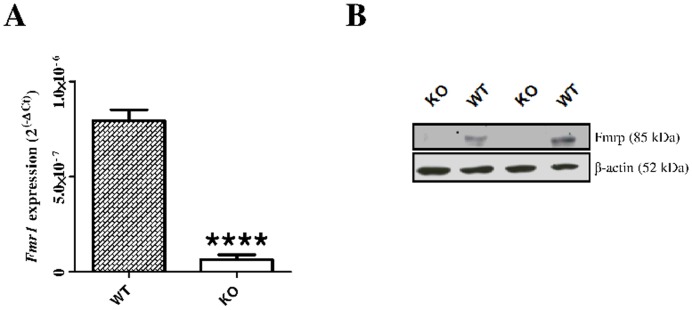
*Fmr1/*Fmrp expression in WT and *Fmr1* KO retinas. (**A**) *Fmr1* mRNA expression was quantified by qPCR (n = 8 per group). Data are expressed as 2^−ΔCt^ values and normalized to 18S RNA internal control. Significant expression of *Fmr1* was only found in WT retina. (**B**) Fmrp expression was assessed by Western-blot analysis (n = 8 per group). All protein variations were normalized to β-actin. Fmrp was present in WT retinas whereas no detectable protein was observed in *Fmr1* KO. Data are presented as Mean ± SEM. Three independent experiments were performed with similar results. Student’s t test, ****p<0.0001.

Quantitative PCR and Western-blot showed that *Fmr1* (Fig. 1A) and its coded protein Fmrp (Fig. 1B) are expressed in the WT retina. In *Fmr1* KO mice, no significant mRNA or protein were observed, as expected (Fig. 1A, 1B).

### Retinal function is altered in *Fmr1* KO mice

**Figure 2:**
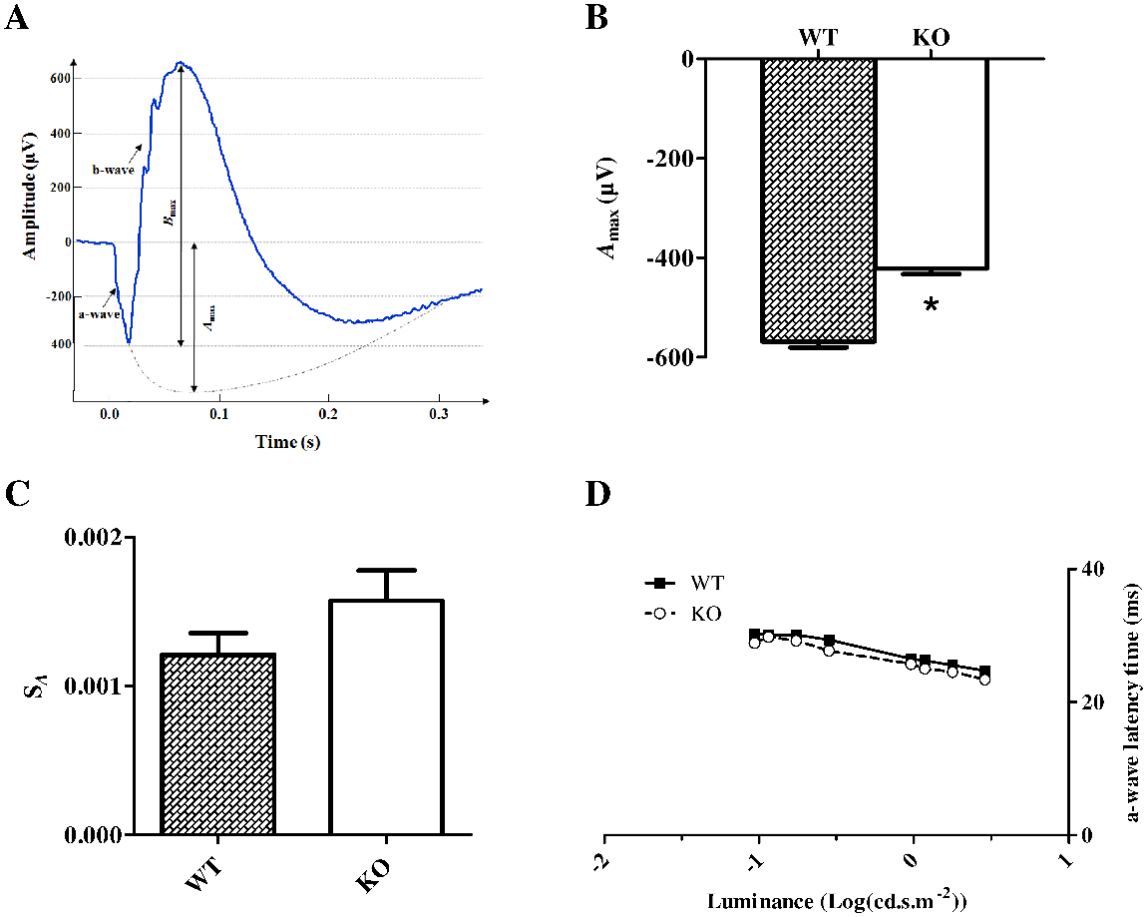
Scotopic a-wave characteristic in WT and *Fmr1* KO mice. Retinal function was evaluated using to ElectroRetinoGram (ERG) (n = 20 for WT and n = 17 for *Fmr1* KO). For each (**A**) typical ERG obtained at light intensity −2.88 log(cd.s.m^−2^), the decreasing part of the a-wave is fitted to calculate the extrapolated (**B**) maximal a-wave amplitude (*A*_max_) and (**C**) S*_A_* parameter reflecting the photoreceptor sensitivity. In *Fmr1* KO mice compared to WT mice, *A*_max_ is significantly decreased by 26% (p<0.05), and the S*_A_* parameter is not significantly different (p = 0.08). (**D**) a-wave latency was not different between groups. Data are presented as Mean ± SEM. Student’s t test, *p<0.05.

In order to evaluate the effect of Fmrp defect on retinal function, we recorded the electrophysiological response of the retina to a light stimulation, called ElectroRetinoGram (ERG). Typically, an ERG is characterized by a negative deflection termed the a-wave, which is initiated by the activity of light-sensitive cells, the photoreceptors (Fig. 2A, Fig. 1S in Supporting Information S1). The following positive deflection, termed the b-wave, reflects signal transmission to the inner retina (Fig. 2A, Fig. 1S in Supporting Information S1), mainly due to bipolar and Müller Cells activities [38]. The small ripples on ascending part of the b-wave, called oscillatory potentials, involved multiple components, presumably including outer and inner retinal circuitry [38].

For each ERG, at the highest light stimulus, the decreasing part of the a-wave was fitted to calculate the maximal a-wave amplitude (*A*_max_) and the parameter S*_A_* reflecting photoreceptor sensitivity. *A*_max_ vary from −568±55 µV in WT mice to −354±43 µV in *Fmr1* KO mice (Fig. 2B) indicating that its amplitude was significantly (p = 0.027) decreased by 26%, whereas S*_A_* was not significantly affected (Fig. 2C). The a-wave latency was not different between WT and *Fmr1* KO mice (Fig. 2D). These observations demonstrated that *Fmr1* KO retinas have a lower photoreceptor response without modification of photoreceptor sensitivity to light.

**Figure 3:**
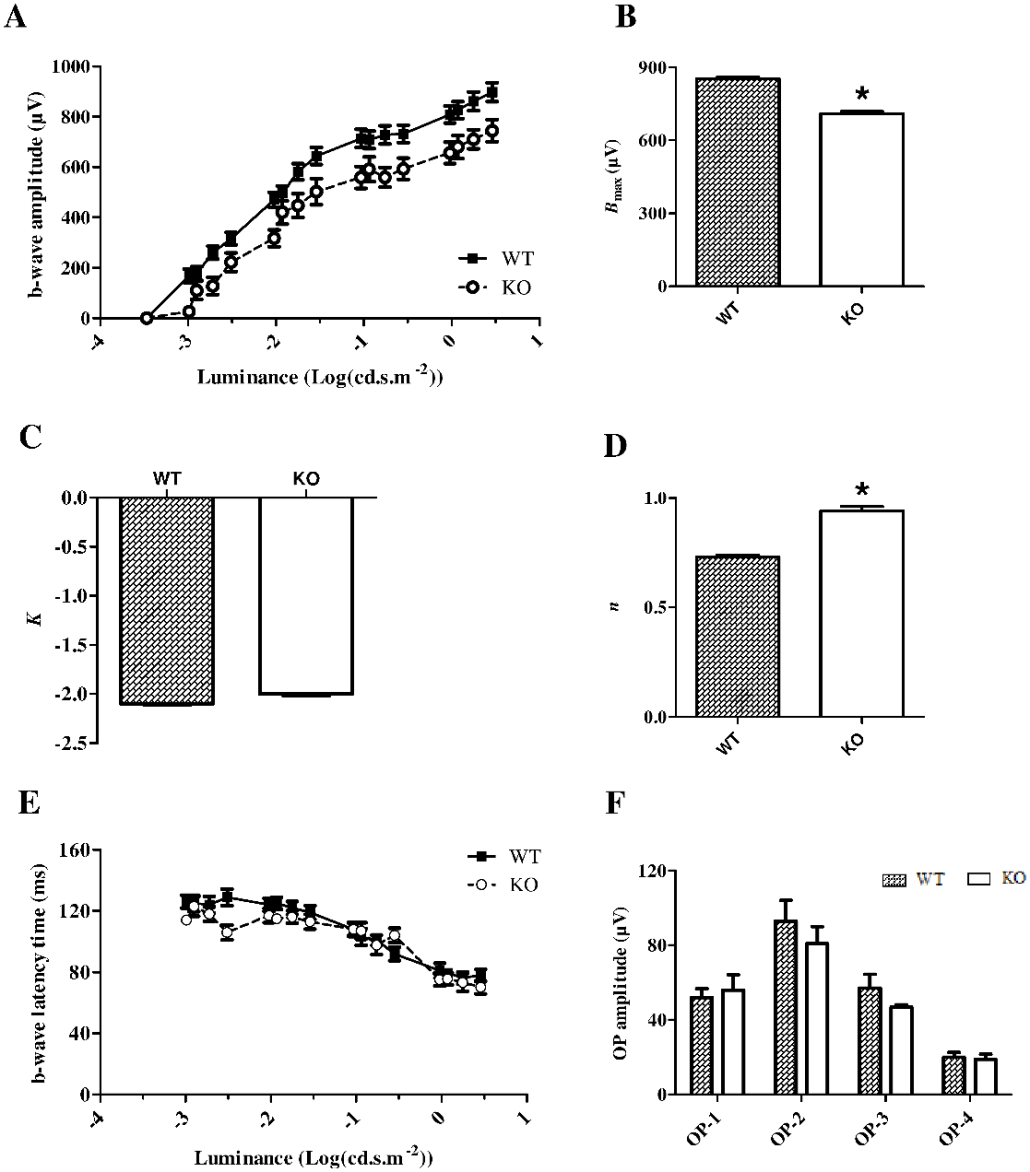
Scotopic b-wave characteristic in WT and *Fmr1* KO mice. Retinal function was evaluated using ElectroRetinoGram (ERG). (**A**) Serial responses to increasing flash stimuli (−3.47 log(cd.s.m^−^^2^) to 0.6 log(cd.s.m^−^^2^)) were obtained for WT and *Fmr1* KO mice under dark-adapted conditions (n = 20 for WT and n = 17 for *Fmr1* KO). ERG response (µV) was plotted against light intensities to obtain the b-wave sensitivity curve. In *Fmr1* KO mice, the b-wave sensitivity curve is slightly collapsed compared to WT mice. The b-wave sensitivity curve was then fitted to calculate (**B**) the saturated b-wave amplitude (*B*_max_), (**C**) the *K* parameter (intensity providing half saturation) and (**D**) the *n* parameter (representing the b-wave sensitivity curves slope). In *Fmr1* KO mice, *B*_max_ is significantly decreased by 20%, the *n* parameter is significantly increased by 29%, whereas *K* parameter remained unchanged compared to WT mice. (**E**) b-wave latency and (**F**) oscillatory potential (OP) amplitude were not different between groups. Data are presented as Mean ± SEM. Student’s t test, *p<0.05.

For each ERG (Fig. 3A), the b-wave sensitivity curve was fitted to calculate the maximal b-wave amplitude (*B*_max_) reflecting the maximal retinal response, the half saturation luminance (*K*) reflecting the light intensity generating half *B*_max_, and the slope of the curve in its linear part (*n*) reflecting the contrast sensitivity of retina. The mean b-wave sensitivity curve of *Fmr1* KO mice was slightly lower than the one from WT mice (Fig. 3A) leading to a significant decrease (p = 0.0085) of *B*_max_ from 853±33 µV in WT mice to 709±39 µV in *Fmr1* KO mice (Fig. 3B). No significant alteration of *K* was observed (Fig. 3C), whereas there was a significant (p = 0.02) increase of *n* from 0.73±0.04 in WT mice to 0.94±0.08 in *Fmr1* KO mice (Fig. 3D). Neither b-wave latency (Fig. 3E) nor the oscillatory potentials (Fig. 3F) were affected in *Fmr1* KO mice. Therefore, *Fmr1* KO retinas have a lower maximal b-wave amplitude and an increase in the slope of the curve in its linear part.

Interestingly, the ratio *B*_max_/*A*_max_ was similar between WT (−1.63±0.13 AU) and *Fmr1* KO (−1.64±0.19 AU) mice, suggesting that the decrease of the maximal b-wave amplitude is mostly due to the decrease of the maximal a-wave amplitude. However, the increase in the slope of b-wave sensitivity curve (Fig. 3D) indicates that a given increase of light-stimulation induced a higher increase of the b-wave in *Fmr1* KO than in WT mice.

All these data demonstrated that *Fmr1* KO mice have an alteration of retinal function characterized by a reduction of the maximal photoreceptor response and an alteration of signal transmission between photoreceptors and the inner retina leading to an increased sensitivity to contrast.

### Retinal synaptic structure is altered in *Fmr1* KO mice

**Figure 4:**
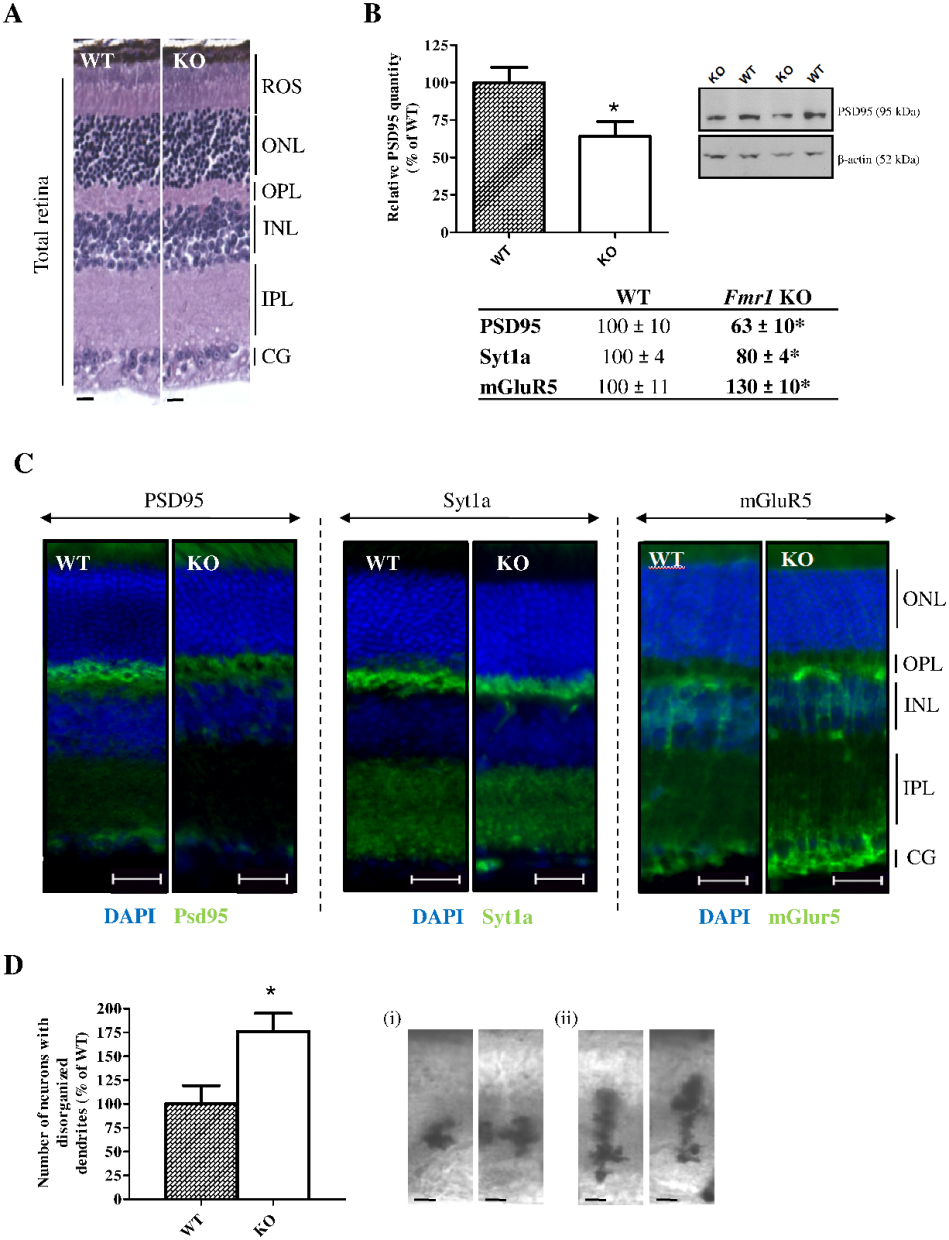
Characterization of neuronal synaptic structure and neuronal immaturity. (**A**) Retinal layer structure was investigated thank to histology. No significant variation of layer thicknesses was observed. Scale bar, 15 µm. (**B**) Retinal synaptic structure was investigated through pre- and post-synaptic markers in WT and *Fmr1* KO retinas. PSD95, Syt1a and mGluR5 expressions were assessed by Western-blot analysis (n = 8 per group). Proteins variations were normalized to β-actin and data are expressed as percentage of WT. PSD95 and Syt1a proteins were decreased by 37% and 20% (*p<0.05) respectively in *Fmr1* KO mice compared to WT retinas whereas mGluR5 was upexpressed by 30% (*p<0.05). Three independent experiments were performed with similar results. (**C**) PSD95, Syt1a and mGluR5 immunolocalisations were realized in *Fmr1* KO and WT retinas (n = 4 per group). PSD95 showed a labelling in Outer Plexiform Layer (OPL), Inner Nuclear Layer (INL) and Ganglion Cells Layer (GCL) for WT retinas. In *Fmr1* KO retinas, all staining was weaker. Syt1a showed a labeling in the OPL for WT retinas as for *Fmr1* KO retinas. mGluR5 showed a labeling in OPL, INL, the Inner Plexiform Layer (IPL) and GCL for WT retinas and for *Fmr1* KO retinas. Scale bar, 20 µm. (**D**) Retinal synaptic immaturity was investigated through Golgi staining. Number of (i) mature neurons and (ii) immature neurons presenting exuberant and disorganized dendrites were counted on 100 µm of WT and *Fmr1* KO retinas and presented as percentage of WT. Number of immature neurons presenting exuberant and disorganized dendrites was significantly (p = 0.046) increased in *Fmr1* KO (176±27%) compared to WT (100±27%). Data are presented as Mean ± SEM. Student’s t test, *p<0.05.

We firstly investigated global retinal histology by measuring retinal layer thicknesses. These thicknesses were not different between WT and *Fmr1* KO mice whatever was the considered layer: ROS (Rod Outer Segment), ONL (Outer Nuclear Layer), OPL (Outer Plexiform Layer), INL (Inner Nuclear Layer) or Total Retina (Fig. 4A, Fig. 2S in Supporting Information S1).

Secondly, to investigate synaptic structure, we focused on Synaptotagmin1a (Syt1a - pre-synaptic) [18], metabotropic glutamate receptor 5 (mGluR5 - post-synaptic) [39], and Post-Synaptic Density protein 95 (PSD95 - post-synaptic) [41]–[43] expressions since their deregulation had been associated with synaptic structure alteration. No variation was observed between WT and *Fmr1* KO retinas for *Syt1a*, *Psd95* and *mGluR5* mRNA (Fig. 3S in Supporting Information S1). In *Fmr1* KO, PSD95 and Syt1a proteins were significantly (p<0.02) down-regulated by 37±10% and 20±4% respectively, compared to WT retinas (Fig. 4B). PSD95 was regularly localised in *Fmr1* KO retina but its staining appeared weaker in the ONL, INL and GCL than for WT retinas (Fig. 4C). Similarly, Syt1a was expressed in the same retinal layers in WT and *Fmr1* KO mice, but the staining seemed more smoothed in *Fmr1* KO mice (Fig. 4C). mGluR5 was expressed in the same layers (OPL, INL, IPL and GCL) in WT as in *Fmr1* KO (Fig. 4C). In the INL, mGluR5 labeling formed vertical streaks that were clearly more intense in *Fmr1* KO retinas compared to WT. This was associated with a significant (p = 0.01) increased from 100±11% in WT to 130±10% in *Fmr1* KO retina of the mGluR5 expression (Fig. 4B).

Thirdly, we investigated synaptic immaturity through Golgi staining. In WT as in *Fmr1* KO retinas, Golgi-stained cells were amacrine cells mainly localized in the INL. However, number of immature neurons presenting exuberant and disorganized dendrites was significantly (p = 0.046) increased in *Fmr1* KO (176±27%) compared to WT (100±27%) (Fig. 4D).

Therefore, the absence of Fmrp had no impact on gross retinal structure suggesting a similar number of retinal neurons in *Fmr1* KO and WT retinas. However, synaptic connections are disrupted. Indeed, deregulation of key pre- and post-synaptic markers, as well as the increased proportion of retinal neurons with exuberant dendrites, clearly evidence synaptic structure alteration and immaturity of retinal neurons in *Fmr1* KO mice.

### Rhodopsin content is decreased in *Fmr1* KO mice

**Figure 5:**
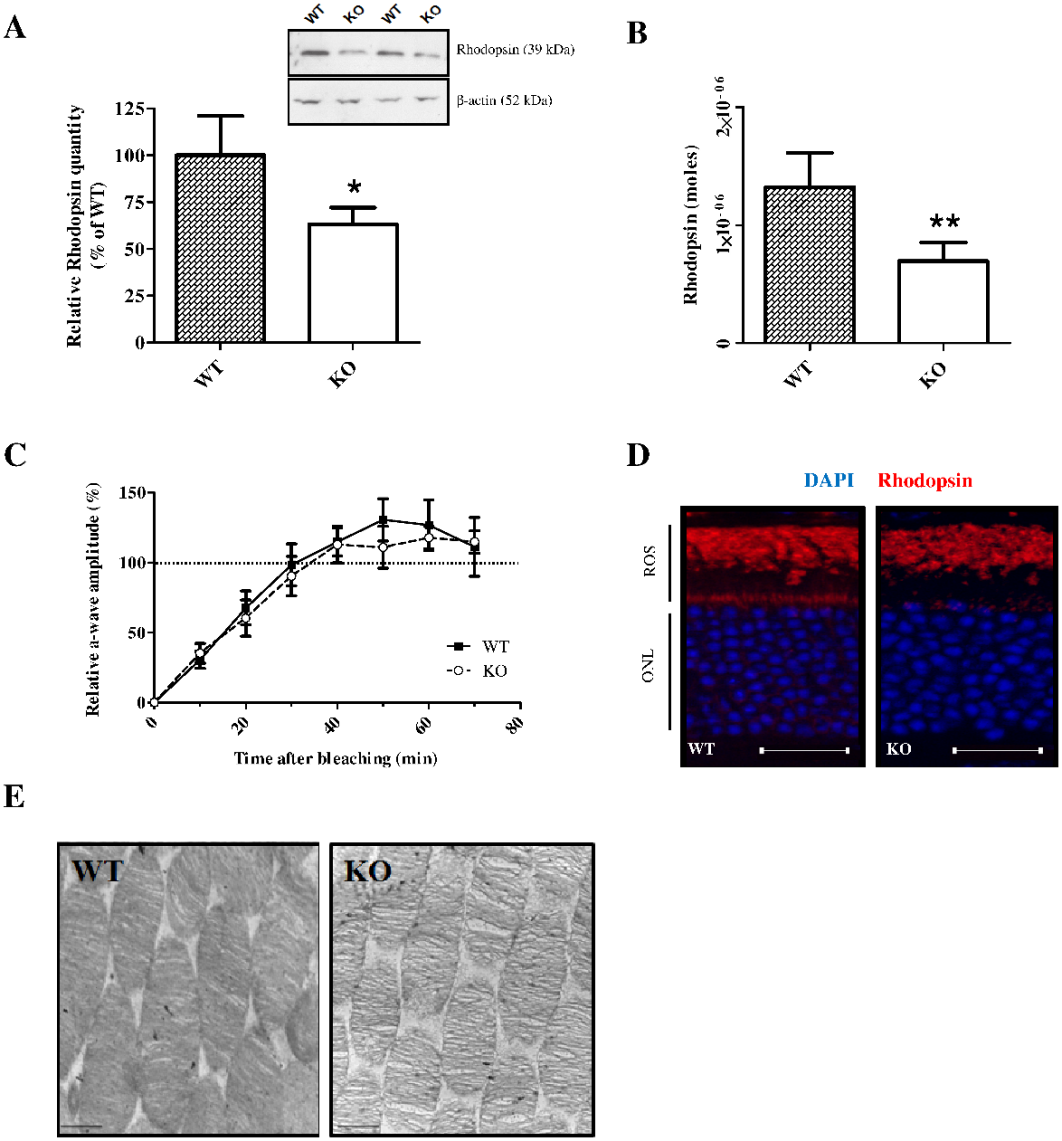
Rhodopsin characterization in adult WT and *Fmr1* KO mice retinas. (**A**) Rhodopsin protein amount was assessed by Western-blot analysis (n = 12 per group). All protein variations were normalized to β-actin. Rhodopsin protein was decreased by 37% (*p<0.05) in *Fmr1* KO mice compared to WT retinas. Three independent experiments were performed with similar results. (**B**) Rhodopsin protein decrease is confirmed by spectrophotometric quantification (n = 6 per group). Indeed, Rhodopsin amount was decreased by 47% (**p<0.01) in *Fmr1* KO mice compared to WT retinas. (**C**) Rhodopsin isomerisation was investigated by studing a-wave recovering following 2 minutes bleach (n = 5 per group). Forty minutes after bleaching the relative a-wave amplitude was returned to dark-adapted value. No difference was observed between WT and *Fmr1* KO retinas. (**D**) Localization of Rhodopsin was done by immunostaining. Immunohistochemistry analysis demonstrated that Rhodopsin is distributed in ROS and between nucleus in the ONL in WT mice, whereas in *Fmr1* KO mice only the ROS labelling could be observed (n = 4 per group). Scale bar, 40 µm. (**E**) ROS ultrastructure was investigate by Transmission Electron Microscopy in adult WT and *Fmr1* KO mice retinas. The images were set at 16000X magnification, with scale bars indicating 50 nm. These representatives pictures showed that density and linear organization of disks in *Fmr1* KO mice ROS are altered compared to WT mice ROS. Data are presented as Mean ± SEM. Student’s t test, *p<0.05, **p<0.01.

In order to better understand the origin of the lower photoreceptor response, we measured Rhodopsin (photopigment) content. Indeed, Rhodopsin is a light-sensing G protein-coupled receptor localised in the membrane of rod photoreceptors that is responsible for light absorption. No difference was observed between WT and *Fmr1* KO retinas for *Rhodopsin* mRNA expression (Fig. 3S in Supporting Information S1). In *Fmr1* KO retinas, Rhodopsin protein content was significantly (p<0.04) decreased by 37±9% as shown by Western-blot analysis (Fig. 5A) and by 47% as shown by spectrophotometric dosage (1320±28 pmoles/retina in WT mice *vs* 696±15 pmoles/retina in *Fmr1* KO mice) (Fig. 5B). The remaining Rhodopsin protein showed an usual recovery after bleaching (Fig. 5C).

Immunohistochemistry analysis demonstrated that Rhodopsin is distributed in ROS and between nucleus in the ONL in WT mice, whereas in *Fmr1* KO mice only the ROS labelling could be observed (Fig. 5D). Although immunohistochemistry is not a quantitative method, we noticed that Rhodopsin staining in *Fmr1* KO was weaker than in WT. Interestingly, although the length of ROS was fairly similar between WT and *Fmr1* KO (data not shown), density and linear organisation of disks in *Fmr1* KO-ROS were clearly altered (Fig. 5E).

Thus, we could show that in *Fmr1* KO retinas, the Rhodopsin content is decreased and ROS structure is altered but Rhodopsin recovery after bleaching is not affected.

## Discussion

The starting point of vision is the detection of light by the retina and more specifically the absorption of light by photoreceptors cells photopigment. In these cells, light is transformed into an electrophysiological signal by a process named phototransduction. This electrophysiological signal, after going through the retina and the optic nerve, reaches the brain and is integrated. Surprisingly, although visual sensory impairments were described in Mental Deficiency (MD) and Autism Spectrum Disorders (ASD), including Fragile X Syndrome (FXS) [22], [23], no data had been collected on light perception at the retinal level even if the retina is a neural tissue with the same embryonic origin as diencephalon [44]. It is even more interesting since our experiments showed that Fmrp is expressed in the WT retina. Therefore, we hypothesized that a lack of Fmrp could induced similar cellular and functional defects in the retina as it does in cerebral neurons.

Retinal function, recorded by ElectroRetinoGram (ERG), is defined by all the electrophysiological manifestations between Rhodopsin activation by light and the electrophysiological message sent through the optic nerve to the brain [38], [45], [46]. As expected, *Fmr1* KO mice showed altered ERG recordings characterized by a decrease in the a and b waves, and an increase in the slope of the b-wave sensitivity curve. These data indicate retinal impairments in *Fmr1* KO mice. Because the *B*_max_/*A*_max_ ratio was similar between *Fmr1* KO and WT mice, we can assume that the b-wave decrease and so the amplitude of the signal transmitted from the photoreceptors to the inner retina is mainly due to the decrease of the a-wave. In addition, a-wave reduction was not due to a loss of photoreceptors, since the ONL thickness was similar between *Fmr1* KO and WT mice, but linked to decreased in Rhodopsin content as shown by Western-blot and spectrophotometric analysis. Indeed, Rhodopsin is the specific rod-photoreceptor protein responsible for the first events in the perception of light [47], and its concentration is directly correlated to a-wave amplitude [38], [48]. In *Fmr1* KO retinas, Rhodopsin was normally localized in the ROS but displayed a ∼40% concentration reduction which results in a 26% reduction in the a-wave. Similar results had already been described in heterozygote *Rhodopsin* knockout mice characterized by a ∼20% *A*_max_ decreased associated with a ∼40% reduced Rhodopsin content [37], [49]. Although we did not notice any reduction of the ROS thickness in *Fmr1* KO retinas, in opposition to Liang et al. study [49], the electronic microscopy showed disorganized and reduced disk density in the photoreceptors outer segment. The lack of Rhodopsin might participate to the destabilization of outer segment [37], [49]. These results raised the question: is the *Rhodopsin* mRNA a target of Fmrp? Further experiments should help to identify other Fmrp targets involved in organ specific impairments.

Electrophysiological data also revealed that *Fmr1* KO mice present an increased slope (*n*) of the b-wave sensitivity curve. This increase indicates that a given raise in light-stimulation induced a higher raise in retinal response which can be interpretable as an alteration in contrast sensitivity/perception. There was no significant difference in the slope for the a-wave amplitude (data not shown) indicating that the alteration in contrast sensitivity/perception could be linked to the transmission of the signal between the photoreceptors and the inner retina rather than an alteration of phototransduction. To explore further this possibility, we looked at pre- (Syt1a) and post- (PSD95 and mGluR5) synaptic markers expression. Indeed, Fmrp is a RNA-binding protein specifically regulating dendrite mRNA translation [50]–[52]. In its absence, *Fmr1* KO neurons *in vitro* [19] or *Fmr1* KO hippocampi [18], [40] and in somatosensory cortex [29] *in vivo*, show a deregulation in several pre- and post-synaptic proteins with, as consequences, a destabilization of synapse structure, immaturity of dendrite spines and an alteration of neurons plasticity [15], [53]. In *Fmr1* KO mice retinas, both pre- and post-synaptic proteins were deregulated, Syt1a and PSD95 being down-regulated whereas mGluR5 was up-regulated. The PSD95 down-regulation is striking since it had been previously linked to plasticity defect and loss of neuron - neuron communication [41], [54]. As a consequence of all these retinal protein deregulations, the immature state of retinal neuron development in *Fmr1* KO mice is characterized by an increase proportion of exuberant and disorganized dendrites. These retinal molecular and cellular phenotypes are clearly similar to those previously described in *Fmr1* KO mice brain, such as synaptic alterations [29], [55] or Fmrp-induced synaptic proteins translation deregulation [19], [56]–[58].

Interestingly, PSD95 had been shown to be unchanged in *Fmr1* KO cerebellum and cortex structures [59] whereas it is up-regulated in cortical neurons culture [60] or whole brain (data not shown), and down-regulated in hippocampus [59] or retina. Since *Psd95* mRNA is one of the Fmrp target [61], we can suggest that the absence of Fmrp is leading to a tissue-specific protein translation deregulation as observed by the different profile in retina/hippocampus and cerebellum/cortex. The specific translation defect is even more reinforced since *Psd95* mRNA level was not altered between WT and *Fmr1* KO retinas. Further molecular experiments on transcriptional/translation modulation linked to Fmrp should help to better understand this tissue specific Fmrp translation regulation.

In agreement with FXS phenotypes, MD or ASD, adult *Fmr1* KO mice exhibit many behavioral impairments on both social and cognitive components, characterized by lower levels of social affiliative behaviors or preferences [62]–[64] and by spatial memory impairments [65], [66]. In this study, we demonstrate for the first time that *Fmr1* deficiency affects retinal function. Therefore, this begs the question of how far vision defects are involved in the recorded behavioral impairments of the *Fmr1* KO mice. Indeed, animal behaviors clearly rely on sensorial processing allowing mice to integrate environmental stimuli and to adapt their action. Based on our study, we can suggest that vision impairments on both perception and integration sides should be taken into account in the behavioural analyses of *Fmr1* KO mice.

As a conclusion, we highlighted for the first time in a mouse model of MD and ASD that sensory impairments involved both peripheral perception and central integration defects. Our findings clearly state that the mis-wiring of neuronal connections and synaptic destabilization in the brain as in the retina lead to similar cellular and functional phenotypes. Based on our data, MD and/or ASD patients, and especially FXS patients, should be investigated on their visual perception.

## Supporting Information

Supporting Information S1 Figure 1S: Representative ERGs obtained from WT or Fmr1 KO mice. Figure 2S: Retinal layers thicknesses in WT and Fmr1 KO retinas. Figure 3S: Synaptic markers mRNA expression in WT and Fmr1 KO retinas.
(DOC)
