# Supplementary material for: Visual Sensorial Impairments in Neurodevelopmental Disorders: Evidence for a Retinal Phenotype in Fragile X Syndrome
Source: PLoS One. 2014 Aug 25;9(8):e105996. doi: 10.1371/journal.pone.0105996 (PMC4143372; doi:10.1371/journal.pone.0105996)
Supplement: Supporting Information S1 — Figure 1S: Representative ERGs obtained from WT or Fmr1 KO mice. Figure 2S: Retinal layers thicknesses in WT and Fmr1 KO retinas. Figure 3S: Synaptic markers mRNA expression in WT and Fmr1 KO retinas. (DOC) [file pone.0105996.s001.doc]

**Supplemental Methods**

**Quantitative RT-PCR**

Quantitative RT-PCR were performed using Taqman technologies (Applied technologies) as described previously [34]. Briefly, total RNA was isolated from retinas using Trizol reagent (Ambion, Life technologies), quantified and reverse transcripted (Superscript iii reverse transcriptase, Invitrogen, Life technologies). Real-time PCR reactions were performed in the Mx3005P Agilent (Applied, Life technologies) with fivefold dilution of cDNA, 200 nM of each Taqman primer using the Expression Master Mix (Applied, Life technologies). Data were analyzed by ΔΔCt method and normalized to the reference standard RNA 18S. Each measurement was performed three times. Controls were obtained from four normal individuals.

**Western Blotting**

Proteins were extracted from mice retinas as described previously [35]. Briefly, the two retinas from the same mice were homogenized in RIPA buffer (10mM Tris-HCl, pH 7.6; 1mM EDTA; NaCl 0.15mM; Igepal 1%; SDS 0.2%; supplemented with protease cocktail inhibitors (Pierce, Paris, France)). Protein concentration was determined in the supernatant by BCA protein assay kit (Pierce, Paris, France). Protein samples (15μg) were run on SDS/PAGE gels (8-12%, w/v), transferred to a nitrocellulose membrane, and probed with primary antibodies (anti-Fmrp, 1:1000; anti-Rhodopsin, 1:2000; anti-PSD95, 1:1000; anti-mGluR5, 1:500 and anti-Syt1a, 1:1000) and secondary HRP-antibody (1:4000) were purchased from ABCam (Paris, France) and Sigma-Aldrich (Illkirch, France). Load of equal amounts of protein was checked by mouse anti-beta Actin antibody (1:4000) (ABCam, Paris, France). Quantification of immunoreactive bands was done using ImageJ software. Western-blots were performed three times. Representative results are shown in figures.

**Electron microscopy**

Electron microscopy was realized in “Centre d’Imagerie Cellulaire Santé” (Clermont-Ferrand, France). Eyes were washed in 0.2M Na cacodylate buffer (pH7.4) and fixed 48h at 4°C in 2% glutaraldehyde with 0.5% paraformaldehyde diluted in 0.2M Na cacodylate buffer (pH 7.4). After removing the cornea and lens, samples were washed three-times in Na cacodylate buffer (0.2M pH 7.4), post fixed 1h with 1% OsO_4_ in 0.2M Na cacodylate buffer (pH 7.4) and washed three-times (10 min) in Na cacodylate buffer (0.2M pH 7.4). For Transmission Electron Microscopy (TEM), specimens were dehydrated in graded ethanol (70%, 95%, 100%) and acetone 100%. They were infiltrated with acetone and EPON resin mixture (2:1) for 1h, with acetone and EPON resin mixture (1:1) for 1h, and with acetone and EPON resin mixture (1:2) for 1h. Specimens were embedded in resin overnight at room temperature, and cured 2 days in a 60°C oven. Thin sections (70 nm) were cut using a UC6 ultramicrotome (Leica, Paris, France) and stained with uranyl acetate and Pb citrate. Carbone was evaporated using a CE6500 unit. Specimen sections were observed at 80 kV with a Hitachi H-7650 TEM with Hamamatsu CDD camera, AMT40. All chemical products were from Electron Microscopy Science, and distributed in France by Delta Microscopies.

**Supplemental Figures**


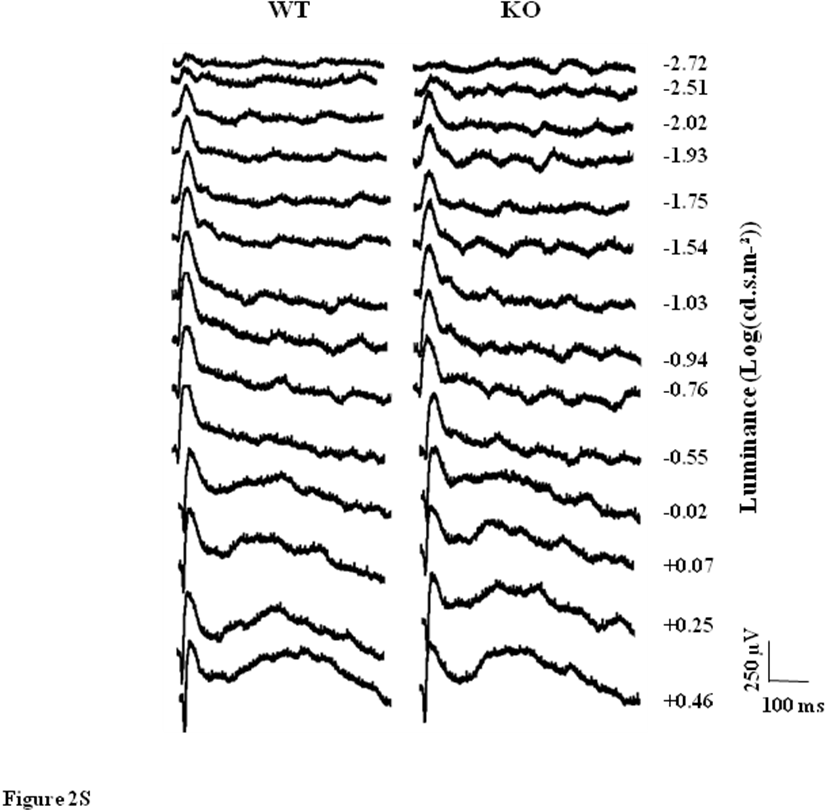


**Figure 1S: Representative ERGs obtained from WT or *Fmr1* KO mice.** WT and *Fmr1* KO mice retinal function were evaluated using ElectroRetinoGram (ERG). Serial responses to increasing flash stimuli (‑2.72 log(cd.s.m^-^²) to 0.46 log(cd.s.m^-^²)) were obtained for WT and *Fmr1* KO mice under dark-adapted conditions (n = 20 for WT and n = 17 for *Fmr1* KO).

**Figure 2S: Retinal layers thicknesses in WT and *Fmr1* KO retinas. (A)** Rod Outer Segment (ROS), **(B)** Outer Nuclear Layer (ONL), **(C)** Outer Plexiform Layer (OPL), **(D)** Inner Nuclear Layer (INL), and **(E)** Total retina thicknesses were measured every 0.76mm from the Optic Nerve (ON) to inferior and superior sides of the retina in WT (*closed squared*) and *Fmr1* KO retinas (*open circles*) (n = 5 per group). No difference was observed between both groups. **(F)** Representative photography of retinal structure, scale bar represents 15 µm.

|  | **WT** | ***Fmr1* KO** |
| --- | --- | --- |
| ***Psd95*** | 1,2E-06 ± 5,6E-07 | 1,4E-06 ± 5,7E-07 |
| ***Syt1a*** | 1,3E-06 ±2,0E-07 | 1,6E-06 ±2,1E-07 |
| ***mGluR5*** | 1,8E-08 ± 2,5E-09 | 1,9E-08 ± 3,6E-09 |
| ***Rhodopsin*** | 1,6E-03 ± 1,0E-04 | 1,8E-03 ± 6,7E-05 |

**Figure 3S: Synaptic markers mRNA expression in WT and *Fmr1* KO retinas.** mRNA expression was quantified by qPCR (n = 8 per group). Data are expressed as 2^-ΔCt^ values and normalized to 18S RNA internal control. No significant difference was observed between WT and *Fmr1* KO retinas. Data (Arbitrary Unit) are presented as Mean ± SEM. Three independent experiments were performed with similar results. Student’s t test, *p<0.05.
